# Multimodality imaging in cardio-oncology: the added value of CMR and CCTA

**DOI:** 10.1259/bjr.20220999

**Published:** 2023-07-26

**Authors:** Costanza Lisi, Federica Catapano, Paolo Rondi, Stefano Figliozzi, Maria Lo Monaco, Federica Brilli, Lorenzo Monti, Marco Francone

**Affiliations:** 1 Department of Biomedical Sciences, Humanitas University, via Rita Levi Montalcini 4, 20072 Pieve Emanuele, Milan, Italy; 2 IRCCS Humanitas Research Hospital, via Manzoni 56, 20089 Rozzano, Milan, Italy; 3 Department of Medical and Surgical Specialties, Radiological Sciences and Public Health, University of Brescia, Brescia, Italy; 4 Cardiology Clinical Department, Faculty of Medicine and Psychology, Sapienza University of Rome, Rome, Italy

## Abstract

During the last 30 years, we have assisted to a great implementation in anticancer treatment with a subsequent increase of cancer survivors and decreased mortality. This has led to an ongoing interest about the possible therapy-related side-effects and their management to better guide patients therapy and surveillance in the chronic and long-term setting. As a consequence cardio-oncology was born, involving several different specialties, among which radiology plays a relevant role. Till the end of August 2022, when European Society of Cardiology (ESC) developed the first guidelines on cardio-oncology, no general indications existed to guide diagnosis and treatment of cancer therapy-related cardiovascular toxicity (CTR-CVT). They defined multimodality imaging role in primary and secondary prevention strategies, cancer treatment surveillance and early CTR-CVT identification and management.

Cardiac computed tomography angiography (CCTA) has acquired a central role in coronary assessment, as far as coronary artery disease (CAD) exclusion is concerned; but on the side of this well-known application, it also started to be considered in left ventricular function evaluation, interstitial fibrosis quantification and cardiac perfusion studies.

Cardiac magnetic resonance (CMR), instead, has been acknowledged as the gold standard alternative to trans-thoracic echocardiography (TTE) poor acoustic window in quantification of heart function and strain modifications, as well as pre- and post-contrast tissue characterization by means of T1-T2 mapping, early Gadolinium enhancement (EGE), late Gadolinium enhancement (LGE) and extracellular volume (ECV) evaluation.

Our review is intended to provide a focus on the actual role of CMR and CCTA in the setting of a better understanding of cardiotoxicity and to draw some possible future directions of cardiac imaging in this field, starting from the recently published ESC guidelines.

## Background and definition

During the last 30 years, we have assisted to a great implementation in anticancer treatment with a subsequent huge increase of cancer survivors.^1,2^ This has led to an ongoing interest about the possible therapy-related side effects and their management to better guide patients therapy and surveillance in the chronic and long-term setting.^3^ For this purpose, a new discipline was born, namely, cardio-oncology^4^, involving several different specialties, among which radiology plays a relevant role in both cardiotoxicity identification and follow-up through multimodality imaging.

Over the past decades, different definitions have been used to describe cancer therapy-related cardiovascular toxicity (CTR-CVT), generating confusion and misdiagnosis. Among them, the most common is the one given by the American Society of Echocardiography, describing cancer treatment-related cardiac dysfunction as a decrease in left ventricular ejection fraction (LVEF) of >10% to a value <53%.^5^ Nowadays, the consensus statement of the International Cardio-Oncology Society (IC-OS)^6^ has overcome these issues with a specific definition of cancer therapy-related cardiac dysfunction (CTRCD) as cardiomyopathy, heart failure (HF), myocarditis, vascular toxicity, hypertension (HTN), cardiac arrhythmias, and corrected QT interval prolongation induced by anticancer therapy. For CTR-CVT-induced pericardial and valvular heart diseases, definitions are the same used for the general population.

Till the end of August 2022, no international guidelines have existed to guide diagnosis and therapy of CTR-CVT, when the first European Society of Cardiology (ESC) guidelines on cardio-oncology were developed in collaboration with the European Hematology Association (EHA), the European Society for Therapeutic Radiology and Oncology (ESTRO) and IC-OS.^7^

As already stated, multimodality imaging plays a central role in primary and secondary prevention strategies, cancer treatment surveillance and early CTR-CVT identification and management. Our review will mainly focus on the actual role of cardiac magnetic resonance (CMR) and cardiac computed tomography angiography (CCTA) in the setting of a better understanding of cardiotoxicity predictive factors, early diagnosis, treatment, and follow-up.^8^

## Cardiotoxicity manifestations and mechanisms

The most common clinical manifestations of CRT-CVT are acute and chronic HF, myocardial ischemia, electrophysiological disturbances, HTN, and vessel thromboembolism.^9^ We will briefly analyze them from a clinical and pathophysiological point of view.

HF is mostly induced by the administration of anthracyclines, cyclophosphamide, ifosfamide, bevacizumab, trastuzumab, and imatinib. From the pathophysiological point of view, free radical formation is recognized as the main mechanism of cardiotoxicity in anthracyclines treatment.^10^ Cyclophosphamide and ifosfamide are thought to cause direct endothelial injury by means of extravasation of toxic metabolites leading to cardiomyocytes damage, interstitial damage, and hemorrhage.^11^ Bevacizumab has been demonstrated to induce HF via uncontrolled HTN and inhibition of Vascular Endothelial Growth Factor (VEGF),^12^ while Trastuzumab interferes with normal cardiomyocytes growth by inhibiting their epidermal growth factor receptor 2 (Erb2).^13^

Myocardial ischemia is commonly diagnosed in cancer patients, particularly in those treated with antimetabolites, antimicrotubules, and monoclonal antibody-based tyrosine kinase inhibitors.^14^ From the pathophysiological point of view, coronary artery thrombosis, arteritis, and vasospasms are proposed as the main causative mechanisms of ischemia in antimetabolites and antimicrotubules therapy (^15^). Bevacizumab, instead, seems to cause myocardial ischemia through its anti-VEGF mechanism.^16^

Bradycardia and QT prolongation are the main electrophysiological disturbances found in CRT-CVT.^14^ They can be caused by anti-cancer-induced fibrosis and radiation therapy, directly affecting the heart conduction system.^17^ Concerning bradycardia, paclitaxel is considered as the main causative agent among chemotherapeutic drugs via its direct effect on Purkinje fibers or extracardiac autonomic control.^18^ QT prolongation causative mechanisms remain unknown and are still quite rare, especially related to small molecule tyrosine kinase inhibitors.

HTN is the most frequent comorbid condition reported in cancer patients.^19^ Studies have shown that it may be related to the VEGF inhibition, decreasing nitric oxide production in arterioles leading to increased vasoconstriction and peripheral vascular resistance.^20^

Cancer patients commonly stay in a pro-thrombotic state, which is in some cases worsened by specific anticancer treatment, like alkylating agents and angiogenesis inhibitors, frequently resulting into vessels’ thromboembolism.^21^ Cisplatin, for example, induces platelet activation and aggregation interfering with monocytes pro-coagulant activity and elevating von-Willebrand factor levels.^22^

Clinical manifestations for chemotherapeutic agents are reported in Table 1.

**Table 1.** A table representing the main CRT-CVT clinical manifestations associated with the most common causative anticancer treatment

Clinical manifestations	Chemotherapeutic agents
Left ventricular dysfunction	Anthracyclines
	Alkylating agents
	Antimetabolites
	Antimicrotubule agents
	Monoclonal antibody-based tyrosine kinase inhibitors
	Small molecule tyrosine kinase inhibitors Proteasome inhibitors
Myocardial ischemia	Antimetabolites
	Antimicrotubule agents
	Monoclonal antibody-based tyrosine kinase inhibitors
	Small molecule tyrosine kinase inhibitors
Myocarditis	Anthracyclines
	Monoclonal antibody-based tyrosine kinase inhibitors
	Immune-checkpoint-inhibitors
Conduction abnormalities	Antimicrotubule agents
	Antiangiogenetic agents
	Histone deacetylase inhibitors
	Small molecule tyrosine kinase inhibitors
HTN	Monoclonal antibody-based tyrosine kinase inhibitors
	Small molecule tyrosine kinase inhibitors
Vessel thromboembolism	Alkylating agents
	Antiangiogenetic drugs
	Histone deacetylase inhibitors
	Small molecule tyrosine kinase inhibitors

### Cardiotoxicitiy in immunotherapy

In the last decades, immunotherapy has become of common use in the treatment of various diseases, such as non-small cell lung cancer, renal cell carcinoma, and both Hodgkin and non-Hodgkin lymphoma. Checkpoint inhibitor immunotherapy (also known as immune checkpoint inhibitors [ICI]) is immunomodulatory antibodies that are used to enhance the immune system. These agents have substantially improved the prognosis for patients with advanced malignancy.

Despite clinical benefit, ICI are associated with a broad spectrum of side effects that are caused mainly by their activity on the immune system: these side effects are known as immune-related adverse events (IrAEs) with various manifestations including cardiotoxicity in up to 1% of patients.^23^

Its manifestation typically includes hypotension, arrhythmia, and left ventricular dysfunction, typically in the setting of cytokine release syndrome.

Of significant interest for the role of advanced imaging is the occurrence of acute ICI induced myocarditis, whose incidence is documented around 0.04–1.14% and which has been associated with a worsening of mortality.^24,25^ Clinical features are extremely variable, in terms of disease onset and severity of manifestations, ranging from fatal myocarditis to transient myocardial edema. Median interval time from first treatment to disease presentation is typically 1–2 months, but cases of delayed onset have been reported up to one year.^26^

The underlying pathophysiology for ICI-associated myocarditis is not yet fully understood.

One possible mechanism is that T cells could target an antigen shared by both the tumor and the heart. Preclinical studies in mouse models have demonstrated that PD-1 protects the heart against T-cell-mediated inflammation and that PD-1-deficient mice develop myocarditis. Similarly, CTLA-4-deficient mice also developed autoimmune myocarditis with infiltration of CD4+ and CD8+ T lymphocytes in the myocardium. Deletion of the CTLA-4 and PD-1 axis led to autoimmune myocarditis, which suggests that PD-1/PDL1 and CTLA-4 play important roles in limiting T-cell-mediated autoimmune myocarditis.

Other mechanisms underlying ICI-mediated cardiotoxicity include pericardial inflammation by ICI-stimulated cytotoxic T-cells and myocardial ischemia. Indeed, ICI-associated inflammation may act on atherosclerotic coronary plaques and trigger fibrous cap rupture, leading to acute myocardial infarction. A second possible underlying mechanism of ICI-associated acute myocardial ischemia is coronary spasm causing ST elevation secondary to PD-1 inhibitor (pembrolizumab) treatment. The exact mechanism of coronary spasm remains unclear but may be related to systemic inflammatory response syndrome. A third explanation for ICI-related acute MI is the direct activation of T-cell-mediated coronary vasculitis in the absence of atherosclerosis. An indirect effect of ICIs on the coronary vasculature via a sudden release of large amounts of catecholamine from the adrenal glands or postganglionic sympathetic nerves in the heart, could result in catecholamine-mediated myocardial stunning (Tako-tsubo syndrome).^27^

Almost all cardiovascular adverse reactions, especially myocarditis, are firstly clinically suspected with cardiac enzymes and ECG alteration, but most of the evidence suggest CV imaging is requested to perform an accurate diagnosis. For this purpose, trans-thoracic echocardiography (TTE) and CMR are interchangeably indicated by 2022 ESC guidelines, but a specific and exclusive role of CMR could be postulated on the basis of modified Lake Louise Criteria accuracy.^28^

## Imaging state of the art

### Insight into ESC 2022 international guidelines

**Figure 1.**
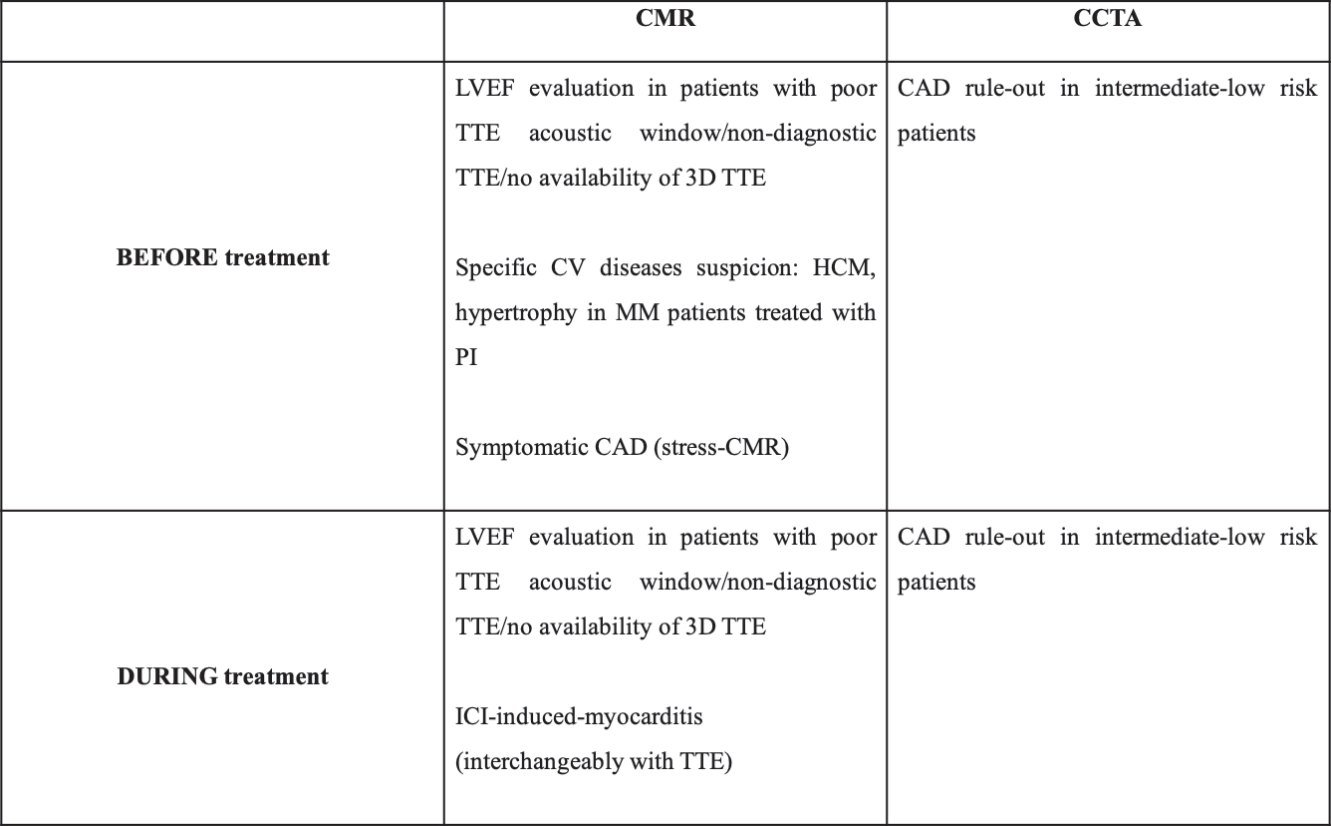
A table summarizing the main indications to CMR and CCTA according to ESC 2022 cardioncology guidelines.

First guidelines in cardioncology were released by ESC on August 2022,^7^, aiming at giving all the healthcare professionals the instruments to take care of oncologic patients before, during and after cancer treatment as far as the cardiovascular system is concerned. They represent a consensus on definition, diagnosis, treatment, and prevention of CTR-CVT. Guidelines include clear indications regarding the role and the appropriate use of different imaging modalities in various clinical scenarios, namely, cardiovascular toxicity risk stratification before anticancer therapy and cardiovascular surveillance during treatment (Figure 1) .

Advanced cardiovascular imaging can be used to identify patients with sub-clinical cardiovascular diseases, directly influencing therapeutic decisions and representing a time zero reference for changes during treatment.^29,30^ ESC guidelines confer a primary role to TTE in the baseline evaluation of heart function and structure prior to chemotherapy,^31^ while CMR role is limited to the evaluation of LVEF in patients with poor acoustic window.

Moreover, whenever TTE is misleading in identifying specific cardiovascular disease, like hypertrophic cardiomyopathy, CMR has to be considered for further risk assessment.

CCTA and/or CMR are also suggested to identify subclinical coronary artery disease (CAD) by means of coronary calcium score quantification and to detect intracardiac masses.^32^ Patients with symptomatic CAD should be tested with functional imaging for myocardial ischemia, like stress-CMR, while in patients with low-intermediate risk of CAD, CCTA represents the standard test to rule out the pathology.^33^

**Figure 2.**
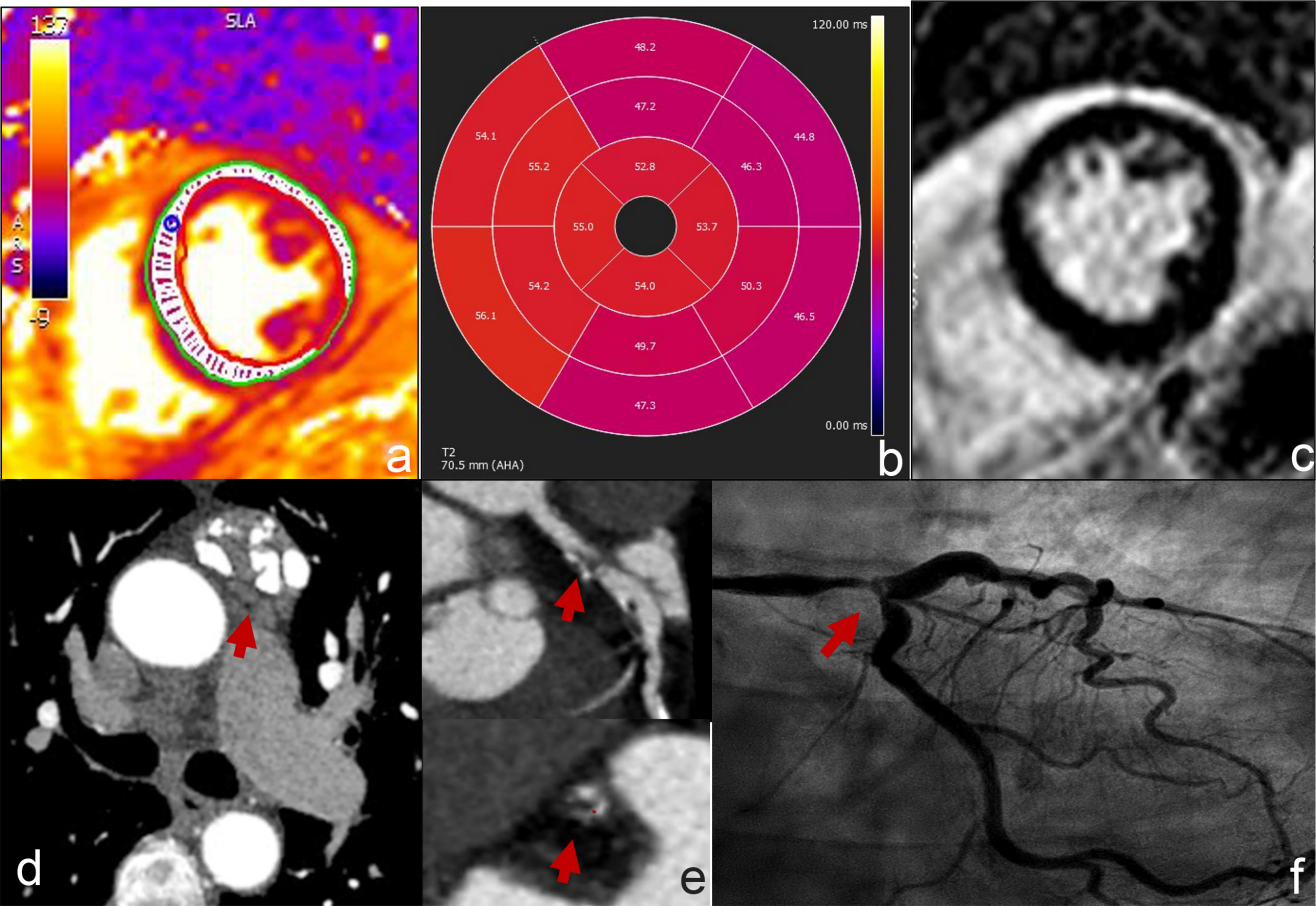
56-year-old female affected by diffuse large B cell lymphoma, treated with ipilimumab-nivolumab, presenting 50 days after treatment with a suspected myocarditis. She underwent CMR showing a focally increased T2 relaxation time in left anterior descending artery territory (a,b) with no evidence of LGE (no myocardial damage) (c). CCTA was performed to rule out CAD, showing a severe stenosis of left main coronary artery (e, red arrow), confirmed at invasive coronary angiography (f, red arrow). CCTA also shows large partially calcified anterior mediastinal lymphoadenopathy as a hallmark of previous radiotherapy (d, red arrow).

Cardiac imaging also exerts a relevant role in decision-making during anti-cancer therapy.^34^ Accurate imaging techniques are requested for this aim, like 3D echocardiography or CMR.^35^ Choice of favourite imaging technique depends on the local availability and expertise; no strict indication favors TTE over CMR at this point but the same imaging modality is recommended over the entire follow-up to allow a precise comparison.^36^ TTE is still recommended for left ventricular function and GLS assessment to detect cardiac dysfunction,^37^ but in case of poor image quality or non-diagnostic TTE, CMR has to be considered^38^ (Figure 2).

A specific indication for CMR exists as far as cardiovascular surveillance for multiple myeloma (MM) targeted therapy is concerned. Proteasome inhibitors (PI) – bortezomib, carfilzomib, and ixazomib – have become a mainstay for MM treatment.^39^ PI are associated with a high incidence of cardiovascular disease (CVD) like HTN, HF, acute coronary syndromes, arrhythmias, pulmonary HTN, and venous thromboembolism.^40–43^ Among them, HF with preserved ejection fraction is frequently associated with cardiac amyloidosis in MM but also to PI therapy; in this setting, a baseline CMR before treatment initiation followed by TTE every three cycles of PI is the commonly accepted surveillance protocol.^7^

### Current imaging role in clinical practice

#### CCTA

CCTA is a widely available imaging technique for the evaluation of coronary anatomy and pathology, which can be exploited in the setting of secondary prevention, as far as CAD rule-out is concerned.

Pre-existing CAD has been demonstrated to contribute to an increased risk of cardiovascular toxicity in patients undergoing anti-cancer treatment.^44^ Despite this knowledge, while clear recommendation exists to rule out CAD in case of first evidence of HF with reduced ejection fraction, no guidelines recommend a pre-treatment evaluation of CAD. Due to its high sensitivity in excluding CAD, CCTA is now widely used to exclude coronary stenosis in patients with exposure to cardiotoxic anticancer treatment and newly developed reduced LVEF.^45^ Non-contrast CT provides information about calcium burden within the coronary arteries as a reliable predictor of cardiovascular risk^46^; it can be used to drive cardio-protective therapy before and during cytotoxic anticancer treatment like anthracyclines as proposed by the society of Cardiovascular Computed Tomography and the Society of Thoracic Radiology.^47^

An example of CAD rule-out by CCTA is shown in Figure 2.

#### CCTA limitations

As already stated, CCTA value in Cardioncology field is nowadays confined to CAD rule-out in case of reduced LVEF, playing a limited role in all the other possible clinical scenarios, according to ESC 2022 guidelines.(^7^) No strong evidences exist about CCTA role in cardiac function assessment and tissue characterization, where CMR is still superior. As far as tissue characterization is concerned, which is the main early markers of cardiotoxicity, future CCTA technology, like Photon Counting CT, may lead to CCTA as a competitive imaging modality in CTR-CVT early recognition and prevention.^48^ Radiation exposure issues still represent a major limitation to CCTA use, although new multidetector machine already maximally reduce dose.^49^

#### CMR – Functional evaluation and tissue characterization

Due to its high spatial and temporal resolution, CMR is a highly accurate modality to measure LVEF without any geometrical assumption. According to the American Society of Echocardiography, CTR-CVT occurs whenever LVEF decreases below normal values (53%) or more than 10% without other recognized causes.^50^ Mendelez et al^51^ studied a cohort of more than 100 patients undergoing potentially cardio-toxic chemotherapy, mainly anthracyclines, and found out that 20% of the population developed a significant drop in LVEF.

The spectrum of tissue abnormalities characterizing myocardial toxic effects of chemotherapy ranges from tissue edema and necrosis to the development of focal replacement and interstitial fibrosis. CMR has also been recognized as a significant prognostic tool in various clinical settings, which can be easily extended to the field of cardio-oncology.^52^

**Figure 3.**
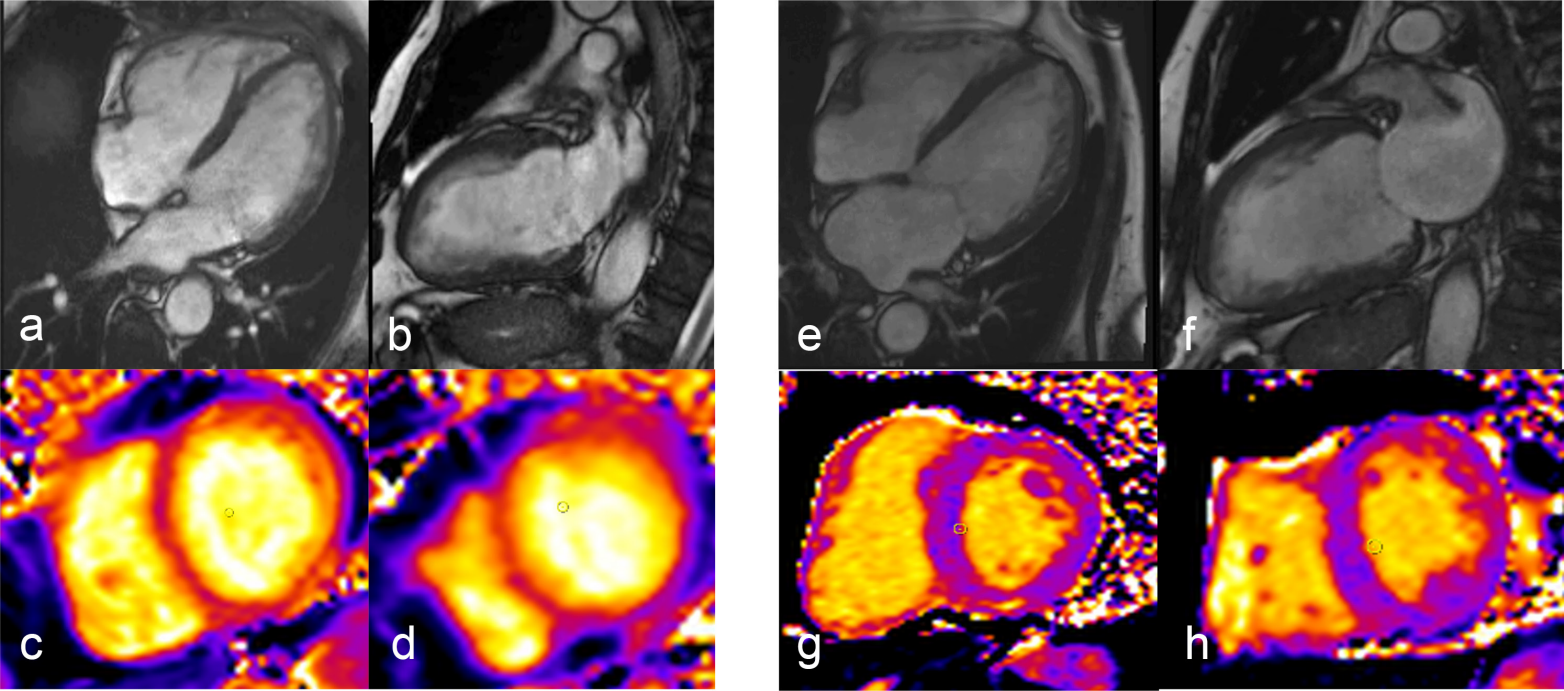
48-year-old female affected by breast carcinoma studied with CMR at baseline and 3 months after anthracycline therapy. 4 and 2 chamber functional evaluation (a,b before therapy and e,f after therapy) show a biventricular function drop and dilation correlated with a change in parametric mapping: c and d, respectively, show normal T1 and T2 parametric values (981 and 47 msec) and g,h chemotherapy induced increase in both T1 and T2 values (1086 and 57 msec).

Use of multiparametric CMR imaging allows to comprehensively explore the various faces and phases of the process, by means of a combination of conventional sequences with more recently developed T1/T2 mapping imaging^36,53^ (Figure 3). These include the quantification of ECV, as an indirect biomarker of tissue fibrosis and interstitial space expansion.

The best approach to evaluate myocardial toxic effects of chemotherapy is represented by 10 use of quantitative imaging techniques. Myocardial tissue mapping is, accordingly, the favourite approach for the quantification of special extent of tissue damage in diffuse disease or in the presence of subtle regional tissue changes, potentially missed with conventional sequences like late gadolinium enhancement (LGE) and *T_2_*-weighted STIR.

**Figure 4.**
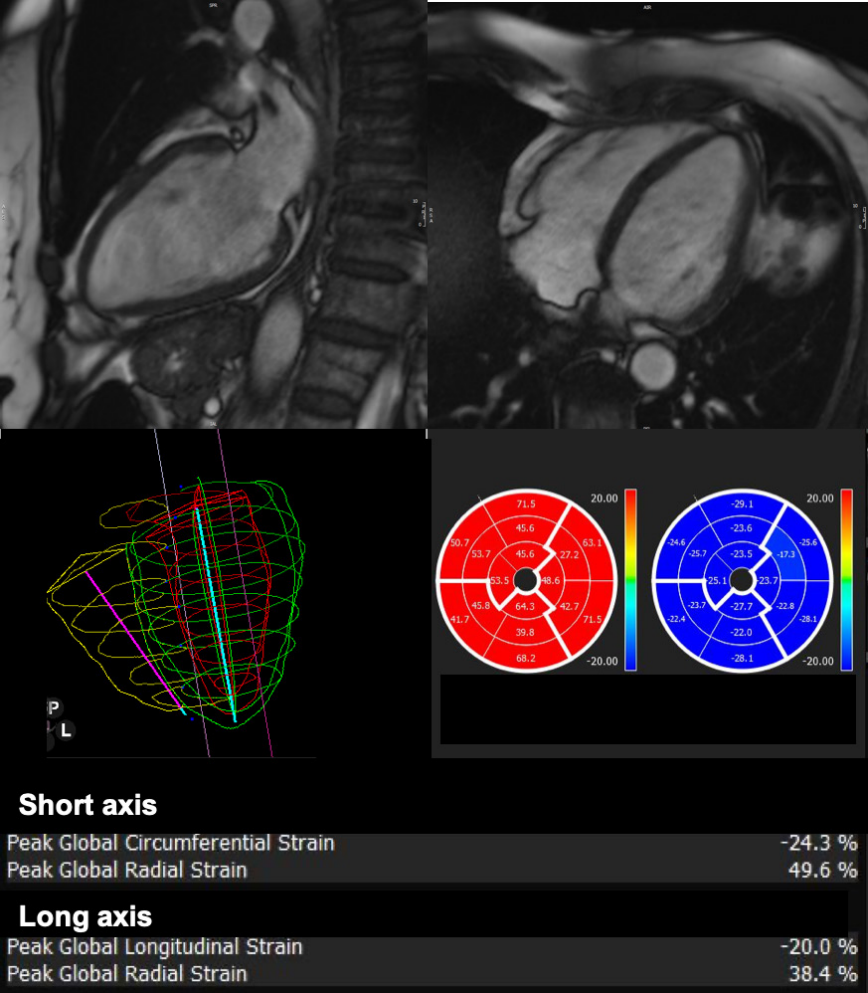
78-year-old female diagnosed with infiltrative breast carcinoma undergoing neoadjuvant chemotehrapy with anthracyclines who underwent CMR after TTE finding of increased trabeculation of the left ventricle. No morphological anomalies were found: LVEF was normal according to age reference values, as you can appreciate from 2 and 4 chamber view in the first row, but strain values were at lower limit of the normal range, as shown in the lower row images.

As a general rule, an increase in pre-contrast T1 is associated with myocardial edema, inflammation, and fibrosis,^54^ while increase in T2 relaxation time is associated with acute myocardial edema, being a water-sensitive process.^55^ Generally speaking, cardiotoxic drug exposure has been associated with an increase in both native T1 and T2 relaxation time^56^ (Figure 4). As an early acute hallmark of cardiotoxicity, many studies stress the pivotal role of increased T2 relaxation time.^57^ Anthracyclines and trastuzumab treatment in HER-2 positive breast cancer patients is associated with an elevated T2 mapping still at a subclinical stage of cardiotoxicity without any LVEF decrease.^58^ Another evidence of early T2 changes as possible hallmark of subclinical cardiotoxicity comes from Lustberg and colleagues, who demonstrated that patients with breast cancer treated with anthracyclines showed no LVEF changes after the first chemotherapy cycle associated with a progressive significant increase in T2 relaxation time over time.^59^ A huge effort has been done to understand and describe temporal evolution of tissue characterization in patients undergoing potentially cardiotoxic chemotherapy. Haslbauer et al^60^ observed early cardiotoxic changes in the form of native T1 and T2 progressive increase during the first month of therapy, followed by a late normalization of T2 time with a permanently elevated non-contrast T1, as a sign of myocardial fibrosis. This work ended up in the definition of an algorithm defining cardiac changes during anticancer treatment and able to detect cardiotoxicity in 84% of cases: early involvement characterized by native T1 >/=2 SD and native T2 >/=2 SD and late involvement with native T1>/=2 SD and normal T2 with or without GLS 17%. More significantly, non-contrast tissue characterization outperformed functional evaluation and GLS in detecting early cardiotoxicity.

Tissue characterization also includes the use of qualitative/semi-quantitative methods based on the administration of gadolinium contrast agents. The presence of early Gadolinium enhancement (EGE) and/or LGE is a hallmark of underlying pathologic processes like inflammation, edema, and myocardial fibrosis^61,62^ also associated with cardiotoxic chemotherapy. More specifically, some studies found EGE to be predictive of LVEF decline after the first month of anthracyclines therapy,^63^ LGE pattern associated with trastuzumab and anthracyclines-induced myocardial fibrosis has been described as non-ischemic-subepicardial contrast enhancement.^64,65^ ECV has the great advantage to detect diffuse myocardial fibrosis even when it is not correctly identified by LGE sequences, as confirmed by a good correlation of ECV values with histological specimens.^66^ ECV increase in the acute setting may be caused by inflammation and interstitial edema^53^ and persistently high ECV values in the long term is reasonable due to edema turning into interstitial fibrosis.^67^

#### CMR limitations

Major CMR limitations derive from its costs and exam tolerability by patients, for which reason TTE still represents the first imaging modality of choice in Cardioncology according to ESC 2022 guidelines.^7^ A limited tolerability of the exam does not allow to perform it in claustrophobic or patients unable to maintain apnea. Despite all the possible application of CMR in function assessment, tissue characterization and strain evaluation, still limited robust evidences exist about their changes in Cardiotoxicity, to allow for accurate diagnosis and prevention of CTR-CVT.

### Potential future imaging role

#### CCTA – ECV quantification

Despite its exclusively approved role in CAD rule-out, literature evidence suggests possible CCTA use for left ventricular function evaluation, cardiac perfusion studies, and tissue characterization.

Multiple studies demonstrated extracellular volume (ECV) increase in patients treated with cardiotoxic chemotherapy compared to matched control population,^68^ and CMR quantification of late gadolinium enhancement was validated as a gold standard non-invasive method for the identification of focal myocardial fibrosis in the setting of ischemic and non-ischemic cardiomyopathy.^69^ In the last 20 years also CCTA has been used for the assessment of cardiac fibrosis by means of ECV quantification. The same imaging protocol as CMR has been adopted with a non-contrast low-dose ECG-gated CCTA acquisition followed by multiple imaging after contrast administration at a given time delay, usually around 10 min.^70^ A good correlation was found between CCTA findings and both CMR ECV and histological fibrosis^71,72^ also in cancer patients undergoing anthracycline chemotherapy regimens.^73^ CCTA-based ECV calculation has also some drawbacks, namely, the increased radiation dose delivered to the patients along with difficulty in image registration and segmentation. These aspects seem to be possibly overcome by the introduction of dual source energy CCT: reduced radiation dose through the elimination of pre-contrast images, improvement of image quality by beam hardening artefact reduction, and less wrong image registration.^74,75^

#### CMR – Functional evaluation with strain

From a functional perspective, subtle early tissue changes following chemotherapy may induce regional wall motion impairments that can be detected using advanced strain CMR-based imaging. This technique is based on the principle of tracking tissue voxel motion using standard steady-state free precession sequences.^76^

In the last years, left ventricular GLS merged as a more accurate index of left ventricular dysfunction, as demonstrated by the SUCCOR (Strain sUrveillance of Chemotherapy for improving Cardiovascular Outcomes) international multicenter prospective randomized controlled trial. Echocardiographic GLS-guided cardioprotective therapy better prevents chemotherapy-induced cardiotoxicity in comparison with LVEF in a high-risk population receiving cardiotoxic chemotherapy.^77^

**Figure 5.**
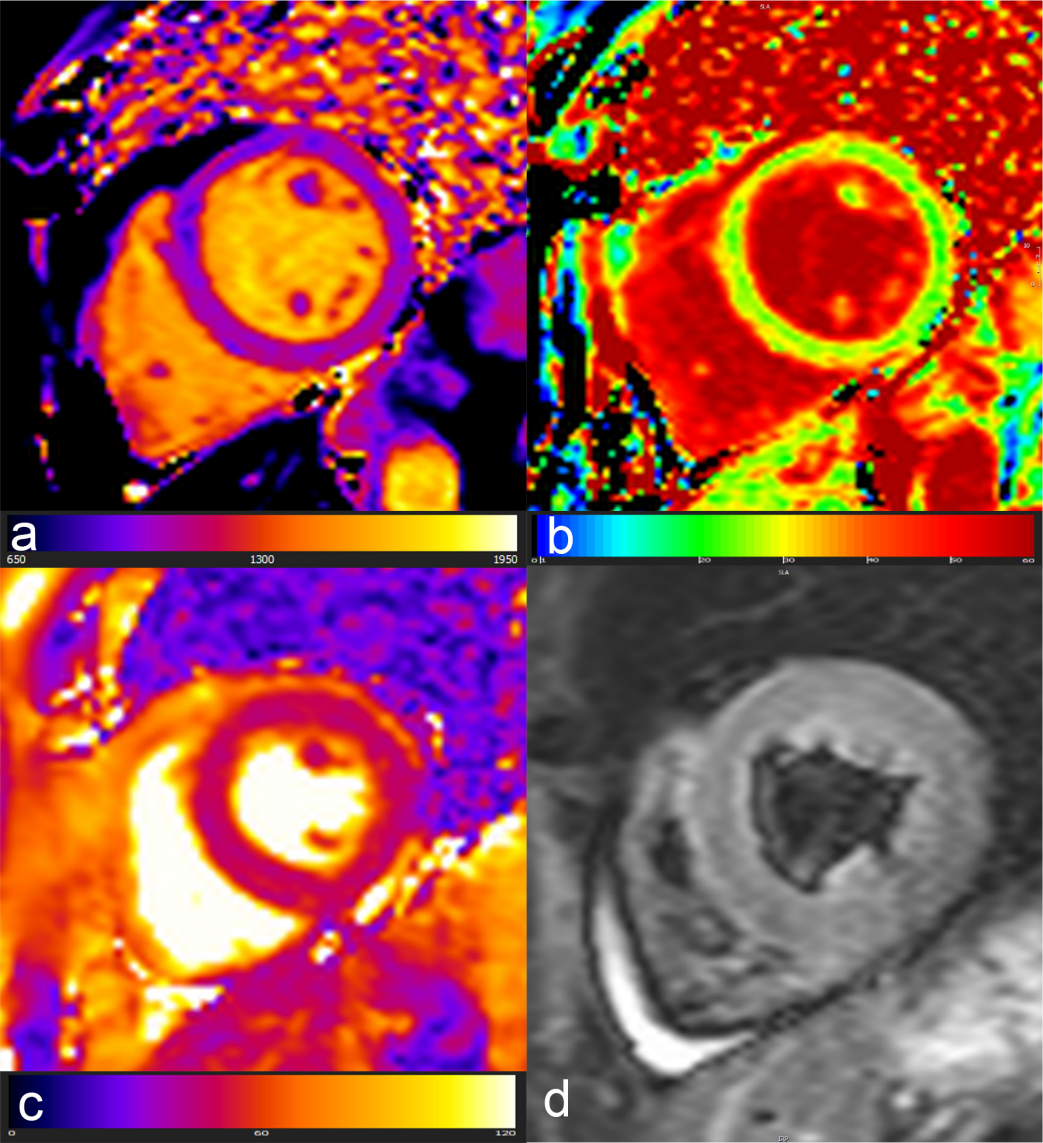
38-year-old female affected by triple negative breast cancer during adjuvant chemotherapy with anthracyclines developing subjective dyspnea. CMR tissue characterization study shows increased native T1 (1040 ms) (a) and ECV (29%) (image b). No edema is found in T2 mapping (c) and inversion recovery sequences (d).

CMR studies have proposed myocardial global longitudinal circumferential and radial strain to describe LV dysfunction, also in patients who have undergone anthracyclines and trastuzumab therapy.^78,79^ A prospective study conducted by Drafts et al^77^ demonstrated an early subclinical deterioration in global circumferential strain (GCS) in patients receiving low-moderate dose of anthracyclines along with subclinical decline of LVEF, proving GCS as an early predictor of cardiotoxicity. An example of the utility of CMR longitudinal circumferential and radial strain as an early marker of cardio-toxicity is shown in Figure 5. CMR can play a central role in early stages, when normal LVEF does not exclude CTR-CVT, and deformation parameters, like GLS, can identify early systolic impairment with good accuracy.^80,81^ Their evaluation is recommended in all patients screened before cardiotoxic cancer treatment initiation to stratify CTR-CVT risk and to identify significant changes during treatment.^82^

Among the different values of CMR strain, GLS and GCS are the most used because of less technical limitations with respect to global radial strain (GRS).^83^

In the specific case of anthracyclines, decline in LVEF and deterioration of strain (GLS, GCS, GRS) values has been postulated to be associated with an atrophic remodeling of myocardium induced by DNA oxidative damage.^84–86^ Coherently, several studies have also demonstrated a significant decrease in left ventricular mass after anthracycline therapy.^87^ De Souza et al^88^ introduced a new method of measuring cardiomyocytes size with CMR, the intracellular water lifetime. It demonstrated a decrease in left ventricular mass due to cardiomyocytes atrophy in females treated with anthracyclines for breast cancer. Another study found an association between left ventricular mass decline, increased risk of cardiac events and HF symptoms more than LVEF decline.^89^

##### CMR and the paradigm of ICI-related myocarditis

Among all possible myocardial inflammation, ICI-related myocarditis represents a specific pathological entity from a clinical, radiological, and histopathological point of view. Differently from non-ICI myocarditis, less than 50% of cases present with a decreased ventricular function and relevant LGE.

Most of patients developing ICI myocarditis show atypical patterns of LGE, without any association with pathology prognosis, meaning LGE approach is necessary but not sufficient for their evaluation.^90^

Quantitative parametric T1 and T2 mapping, as well ECV, seem to represent a real instrument to avoid underdiagnoses and overcome this inconsistency, in such a way to allow for a proper and prompt diagnosis of a pathology which brings a relevant mortality into the general population.^91^

Recent studies, like the one of Paaladinesh et al,^92^ demonstrate a better correlation between T1 and T2 mapping and both clinical and histological ICI-induced myocarditis characteristics. T1 mapping alterations are significantly correlated with symptom severity as well as to the histological specimen; even better and more significantly than T2 mapping, T1 mapping alteration seems to be correlated with the incidence of major cardiovascular adverse events.

**Figure 6.**
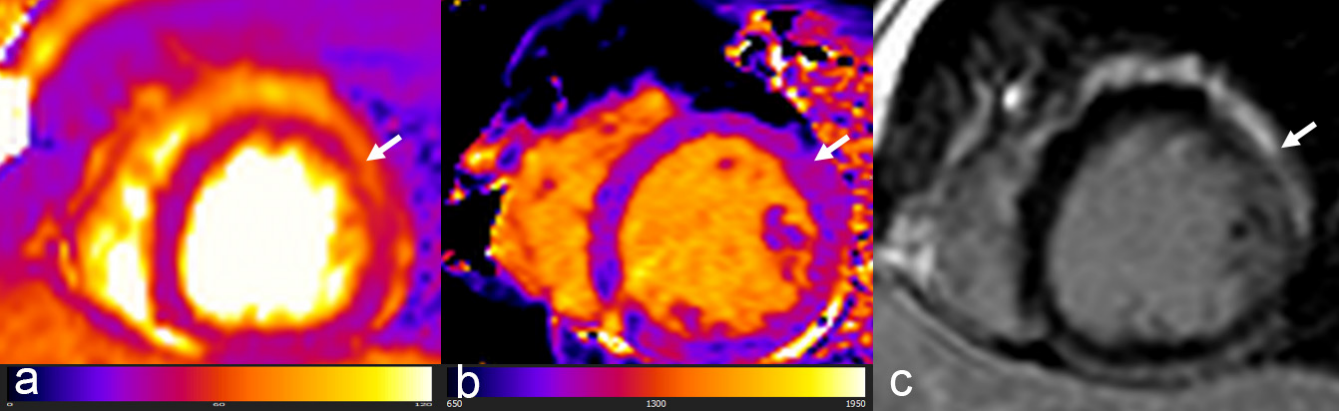
A 35-year-old male affected by metastatic melanoma under treatment with Ipilimumab developing chest discomfort and dyspnea after two weeks of treatment. No relevant ECG changes but increased high-sensitive Troponin was detected. Contrast-enhanced CMR was performed showing increased T2 relaxation time (54.8 msec) in mid anterolateral LV wall (white arrow, (a), consensual increase in T1 relaxation time (1047 msec) (white arrow, (b) and atypical pattern of confluent almost transmural LGE (white arrow, (c), diagnostic for ICI-induced myocarditis.

An example of ICI-induced myocarditis is shown in Figure 6.

This opens to the possibility of designing specific CMR protocols, centered on quantitative parametric mapping, with a diagnostic and prognostic role from the pre-subclinical stages to the full-blown pathology.

## Suggested workflow for CTR-CVT

**Figure 7.**
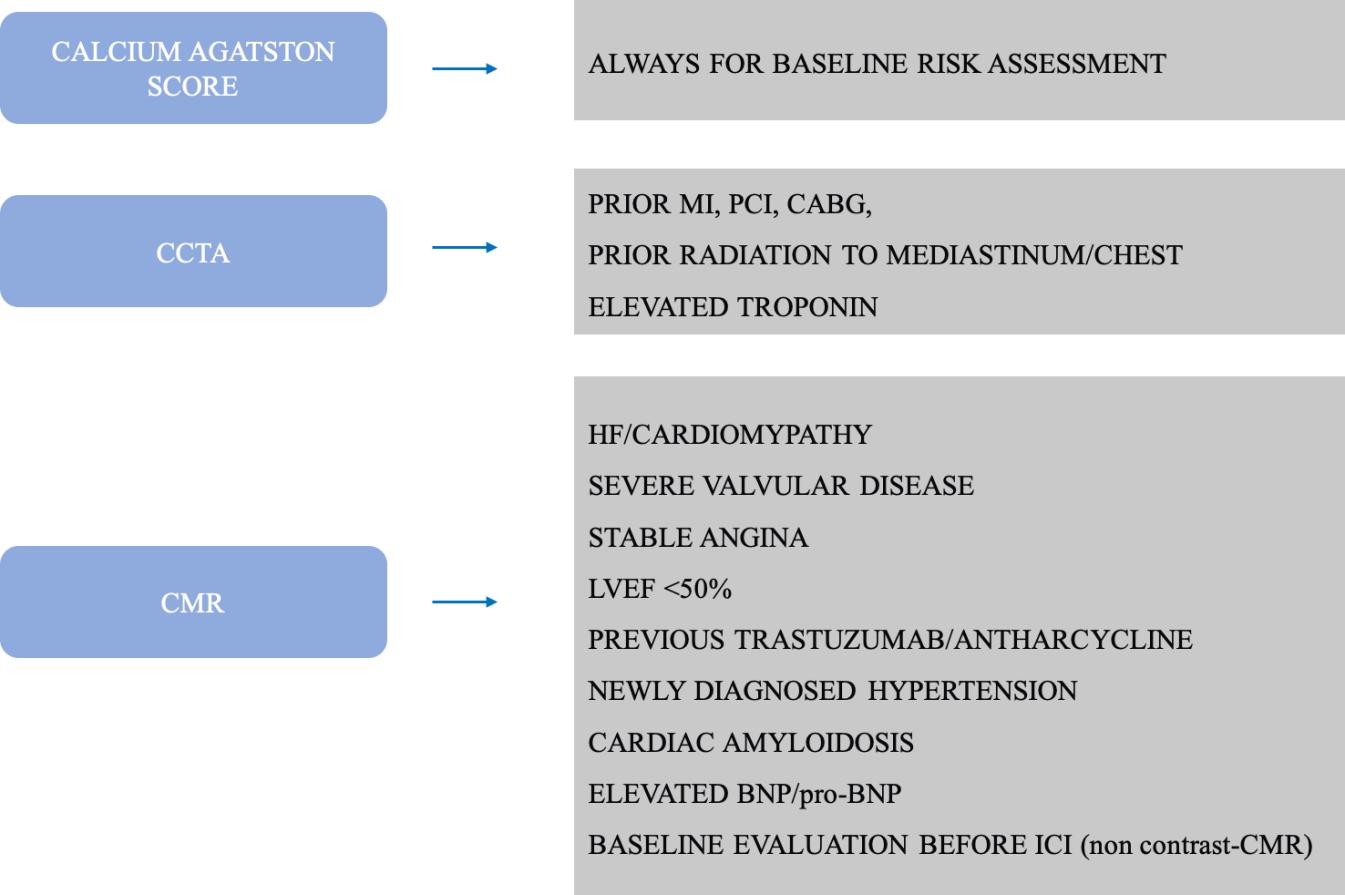
Newly proposed indications to perform advanced cardiac imaging for baseline assessment before potentially cardiotoxic anticancer therapy.

Despite the recently published ESC guidelines, on the basis of an accurate review of the new risk assessment tools from Cardioncology Study Group of the Heart Failure Association of the European Society of Cardiology in collaboration with the International Cardioncology Society,^93^ we propose a novel baseline risk assessment flowchart for patient’s candidate to potential cardiotoxic therapy, according to our Institution’s clinical routine (Figure 7).

A baseline Calcium Score quantification on a non-contrast ECG-gated chest CT performed during the cancer staging phase is strongly suggested according to the strong evidence correlation between coronary calcification and increased risk for a major adverse cardiac event.^94^

## Future perspectives and prognostic implications

Regardless of these solid and consistent scientific bases, current CMR role is apparently limited to patients with poor acoustic TTE window, hypertrophic cardiomyopathy, and PI-induced MM, but its potential goes far beyond these applications.

While TTE is still recognized as the favourite imaging technique for baseline risk stratification,^7^ CMR should be considered as a first-line imaging at least in patients at high/very high risk to develop CTR-CVT, in order to be able to precisely estimate ventricular volumes and function but also to perform tissue characterization and identify subclinical changes by means of mapping, LGE sequences and ECV evaluation. Whenever CMR results normal, patients may be followed-up clinically and according to the standard guideline; in the case of any pathological abnormalities, following investigations should be performed with CMR for the sake of reproducibility and precision.

ESC 2022^7^ guidelines opened a new potential window for CMR application, but the pathway is still ongoing.

CCTA use in this setting still remains limited to CAD exclusion in low-intermediate risk patients^95^ and all other possible applications for functional evaluation, GLS, LGE, and ECV quantification are not even mentioned for its poor availability and standardization. The main potential strength of the introduction of CCTA in cardiovascular toxicity risk assessment and evaluation during therapy is that oncologic patients routinely undergo computed tomography exams for cancer staging, monitoring, and follow-up. This would mean maximizing the usefulness of an exam which cannot be bypassed in oncologic patients and adapting it to a double use: the purely oncologic staging and follow-up but also the possible complete cardiac evaluation in specific subsets of the population at risk of CTR-CVT.

Application of advanced cardiac imaging in oncology is an open field for research.^96^ More evidences are needed about the possible clinical role of the above-mentioned imaging findings in guiding multifactorial therapeutic pathways and decision.

## References

[1] Gevaert SA, Halvorsen S, Sinnaeve PR, Sambola A, Gulati G, Lancellotti P, et al . Evaluation and management of cancer patients presenting with acute cardiovascular disease: a consensus document of the acute cardiovascular care (ACVC) Association and the ESC Council of Cardio-oncology-part 1: acute coronary syndromes and acute Pericardial diseases. Eur Heart J Acute Cardiovasc Care 2021; 10: 947–59. doi: 10.1093/ehjacc/zuab056 34453829

[2] Deo SVS, Sharma J, Kumar S . GLOBOCAN 2020 report on global cancer burden: challenges and opportunities for surgical Oncologists. Ann Surg Oncol 2022; 29: 6497–6500. doi: 10.1245/s10434-022-12151-6 35838905

[3] Zamorano JL, Lancellotti P, Rodriguez Muñoz D, Aboyans V, Asteggiano R, Galderisi M, et al . ESC position paper on cancer treatments and cardiovascular toxicity developed under the auspices of the ESC Committee for practice guidelines: the task force for cancer treatments and cardiovascular toxicity of the European society of cardiology (ESC). Eur Heart J 2016; 37: 2768–2801. doi: 10.1093/eurheartj/ehw211 27567406

[4] Lancellotti P, Suter TM, López-Fernández T, Galderisi M, Lyon AR, Van der Meer P, et al . Cardio-oncology services: rationale, organization, and implementation. Eur Heart J 2019; 40: 1756–63. doi: 10.1093/eurheartj/ehy453 30085070

[5] Plana JC, Galderisi M, Barac A, Ewer MS, Ky B, Scherrer-Crosbie M, et al . Expert consensus for Multimodality imaging evaluation of adult patients during and after cancer therapy: a report from the American society of echocardiography and the European Association of cardiovascular imaging. J Am Soc Echocardiogr 2014; 27: 911–39. doi: 10.1016/j.echo.2014.07.012 25172399

[6] Herrmann J, Lenihan D, Armenian S, Barac A, Blaes A, Cardinale D, et al . Defining cardiovascular toxicities of cancer therapies: an international Cardio-oncology society (IC-OS) consensus statement. Eur Heart J 2022; 43: 280–99. doi: 10.1093/eurheartj/ehab674 34904661 PMC8803367

[7] Lyon AR, López-Fernández T, Couch LS, Asteggiano R, Aznar MC, Bergler-Klein J, et al . ESC guidelines on Cardio-oncology developed in collaboration with the European hematology Association (EHA) the European society for therapeutic Radiology and oncology (ESTRO) and the International Cardio-oncology society (IC-OS). Eur Heart J 2022; 43: 4229–4361. doi: 10.1093/eurheartj/ehac244 36017568

[8] López Fernández T, López Sendón J . Cardiotoxicity. Anti-CancerTreatments and Cardiotoxicity. Elsevier; 2017, pp. 409–11.

[9] Burashnikov A, Abbate A, Booz GW . Cardiovascular complications of anti-cancer therapy: a developing storm in medicine. J Cardiovasc Pharmacol 2022; 80: 491–92. doi: 10.1097/FJC.0000000000001355 36001886

[10] Abrahams C, Woudberg NJ, Lecour S . Anthracycline-induced cardiotoxicity: targeting high-density lipoproteins to limit the damage. Lipids Health Dis 2022; 21(): 85. doi: 10.1186/s12944-022-01694-y 36050733 PMC9434835

[11] Dionísio F, Araújo AM, Duarte-Araújo M, Bastos M de L, Guedes de Pinho P, Carvalho F, et al . Cardiotoxicity of cyclophosphamide’s metabolites: an in vitro metabolomics approach in Ac16 human cardiomyocytes. Arch Toxicol 2022; 96: 653–71. doi: 10.1007/s00204-021-03204-y 35088106

[12] Chen X-Y, Huang W-L, Peng X-P, Lv Y-N, Li J-H, Xiong J-P . miR-140-5p mediates Bevacizumab-induced cytotoxicity to cardiomyocytes by targeting the VEGFA/14-3-3γ signal pathway. Toxicol Res (Camb) 2019; 8: 875–84. doi: 10.1039/c9tx00100j 32190292 PMC7066572

[13] Anjos M, Fontes-Oliveira M, Costa VM, Santos M, Ferreira R . An update of the molecular mechanisms underlying doxorubicin plus Trastuzumab induced cardiotoxicity. Life Sci 2021; 280: 119760. doi: 10.1016/j.lfs.2021.119760 34166713

[14] Yeh ETH, Bickford CL . Cardiovascular complications of cancer therapy: incidence, pathogenesis, diagnosis, and management. J Am Coll Cardiol 2009; 53: 2231–47. doi: 10.1016/j.jacc.2009.02.050 19520246

[15] Li C, Ngorsuraches S, Chou C, Chen L, Qian J . Risk factors of Fluoropyrimidine induced cardiotoxicity among cancer patients: A systematic review and meta-analysis. Crit Rev Oncol Hematol 2021; 162: 103346. doi: 10.1016/j.critrevonc.2021.103346 33930532

[16] Sundararajan S, Kumar A, Poongkunran M, Kannan A, Vogelzang NJ . Cardiovascular adverse effects of targeted Antiangiogenic drugs: mechanisms and management. Future Oncol 2016; 12: 1067–80. doi: 10.2217/fon.16.4 26901457

[17] Tamargo J, Caballero R, Delpón E . Cancer chemotherapy-induced sinus bradycardia: A narrative review of a forgotten adverse effect of cardiotoxicity. Drug Saf 2022; 45: 101–26. doi: 10.1007/s40264-021-01132-5 35025085

[18] Zekri JM . Case report and review of literature: temporary asymptomatic sinus bradycardia with carboplatin, paclitaxel and Bevacizumab: under-reported in clinical trials and under-disclosed in practice. Gulf J Oncolog 2011; 60–64. 21724531

[19] Jain M, Townsend RR . Chemotherapy agents and hypertension: a focus on angiogenesis blockade. Curr Hypertens Rep 2007; 9: 320–28. doi: 10.1007/s11906-007-0058-7 17686384

[20] Kamba T, McDonald DM . Mechanisms of adverse effects of anti-VEGF therapy for cancer. Br J Cancer 2007; 96: 1788–95. doi: 10.1038/sj.bjc.6603813 17519900 PMC2359962

[21] De Stefano V, Larocca A, Carpenedo M, Cavo M, Di Raimondo F, Falanga A, et al . Thrombosis in multiple myeloma: risk stratification, Antithrombotic prophylaxis, and management of acute events. A consensus-based position paper from an ad hoc expert panel. Haematol 2022; 107: 2536–47. doi: 10.3324/haematol.2022.280893 PMC9614522 35861017

[22] Içli F, Karaoğuz H, Dinçol D, Demirkazik A, Günel N, Karaoğuz R, et al . Severe vascular toxicity associated with cisplatin-based chemotherapy. Cancer 1993; 72: 587–93. doi: 10.1002/1097-0142(19930715)72:2<587::aid-cncr2820720242>3.0.co;2-v 8319192

[23] Zimmer L, Goldinger SM, Hofmann L, Loquai C, Ugurel S, Thomas I, et al . Neurological, respiratory, musculoskeletal, cardiac and ocular side-effects of anti-PD-1 therapy. Eur J Cancer 2016; 60: 210–25. doi: 10.1016/j.ejca.2016.02.024 27084345

[24] Lorente-Ros Á, Rajjoub-Al-Mahdi E-A, Monteagudo Ruiz JM, Rivas García S, Ortega Pérez R, Fernández Golfín C, et al . Checkpoint Immunotherapy-induced myocarditis and encephalitis complicated with complete AV block: not all hope is lost. JACC Case Rep 2022; 4: 1032–36. doi: 10.1016/j.jaccas.2022.04.020 36062054 PMC9434642

[25] Jiménez-Alejandre R, Ruiz-Fernández I, Martín P . Pathophysiology of immune Checkpoint inhibitor-induced myocarditis. Cancers 2022; 14(): 4494. doi: 10.3390/cancers14184494 36139654 PMC9497311

[26] Champion SN, Stone JR . Immune Checkpoint inhibitor associated myocarditis occurs in both high-grade and low-grade forms. Mod Pathol 2020; 33: 99–108. doi: 10.1038/s41379-019-0363-0 31534205

[27] Chen D-Y, Huang W-K, Chien-Chia Wu V, Chang W-C, Chen J-S, Chuang C-K, et al . Cardiovascular toxicity of immune Checkpoint inhibitors in cancer patients: A review when cardiology meets Immuno-oncology. J Formos Med Assoc 2020; 119: 1461–75. doi: 10.1016/j.jfma.2019.07.025 31444018

[28] Wintersperger BJ, Calvillo-Argüelles O, Lheureux S, Houbois CP, Spreafico A, Bedard PL, et al . Immune Checkpoint inhibitor-related myocarditis: an illustrative case series of applying the updated cardiovascular magnetic resonance Lake Louise criteria. Eur Heart J Case Rep 2022; 6: 478. doi: 10.1093/ehjcr/ytab478 PMC8783546 35079688

[29] Plana JC, Thavendiranathan P, Bucciarelli-Ducci C, Lancellotti P . Multi-modality imaging in the assessment of cardiovascular toxicity in the cancer patient. JACC Cardiovasc Imaging 2018; 11: 1173–86. doi: 10.1016/j.jcmg.2018.06.003 30092972

[30] Galderisi M, Cosyns B, Edvardsen T, Cardim N, Delgado V, Di Salvo G, et al . Standardization of adult transthoracic echocardiography reporting in agreement with recent chamber Quantification, diastolic function, and heart valve disease recommendations: an expert consensus document of the European Association of cardiovascular imaging. Eur Heart J Cardiovasc Imaging 2017; 18: 1301–10. doi: 10.1093/ehjci/jex244 29045589

[31] Curigliano G, Lenihan D, Fradley M, Ganatra S, Barac A, Blaes A, et al . Management of cardiac disease in cancer patients throughout Oncological treatment: ESMO consensus recommendations. Ann Oncol 2020; 31: 171–90. doi: 10.1016/j.annonc.2019.10.023 31959335 PMC8019325

[32] Phillips WJ, Johnson C, Law A, Turek M, Small AR, Dent S, et al . Comparison of Framingham risk score and chest-CT identified coronary artery calcification in breast cancer patients to predict cardiovascular events. Int J Cardiol 2019; 289: 138–43. doi: 10.1016/j.ijcard.2019.01.056 30696608

[33] Lopez-Mattei JC, Yang EH, Ferencik M, Baldassarre LA, Dent S, Budoff MJ . Cardiac computed tomography in Cardio-oncology: JACC: Cardiooncology primer. JACC CardioOncol 2021; 3: 635–49. doi: 10.1016/j.jaccao.2021.09.010 34988472 PMC8702811

[34] López-Fernández T, Thavendiranathan P . Emerging cardiac imaging modalities for the early detection of cardiotoxicity due to anticancer therapies. Rev Esp Cardiol 2017; 70: 487–95. doi: 10.1016/j.rec.2017.01.004 28189542

[35] Plana JC, Galderisi M, Barac A, Ewer MS, Ky B, Scherrer-Crosbie M, et al . Expert consensus for Multimodality imaging evaluation of adult patients during and after cancer therapy: a report from the American society of echocardiography and the European Association of cardiovascular imaging. Eur Heart J Cardiovasc Imaging 2014; 15: 1063–93. doi: 10.1093/ehjci/jeu192 25239940 PMC4402366

[36] Eschenhagen T, Force T, Ewer MS, de Keulenaer GW, Suter TM, Anker SD, et al . Cardiovascular side effects of cancer therapies: a position statement from the heart failure Association of the European society of cardiology. Eur J Heart Fail 2011; 13: 1–10. doi: 10.1093/eurjhf/hfq213 21169385

[37] Jiang W, Zhao W, Huang H . Strain-guided management of potentially cardiotoxic cancer therapy. J Am Coll Cardiol 2021; 77: 2869–70. doi: 10.1016/j.jacc.2020.12.074 34082917

[38] Korosoglou G, Giusca S, Montenbruck M, Patel AR, Lapinskas T, Götze C, et al . Fast strain-encoded cardiac magnetic resonance for diagnostic classification and risk stratification of heart failure patients. JACC Cardiovasc Imaging 2021; 14: 1177–88. doi: 10.1016/j.jcmg.2020.10.024 33454266

[39] Rajkumar SV . Multiple myeloma: 2020 update on diagnosis, risk-stratification and management. Am J Hematol 2020; 95: 548–67. doi: 10.1002/ajh.25791 32212178

[40] Siegel D, Martin T, Nooka A, Harvey RD, Vij R, Niesvizky R, et al . Integrated safety profile of single-agent Carfilzomib: experience from 526 patients enrolled in 4 phase II clinical studies. Haematologica 2013; 98: 1753–61. doi: 10.3324/haematol.2013.089334 23935022 PMC3815177

[41] Ridolfi RL, Bulkley BH, Hutchins GM . The conduction system in cardiac Amyloidosis. clinical and pathologic features of 23 patients. Am J Med 1977; 62: 677–86. doi: 10.1016/0002-9343(77)90870-1 871125

[42] Fakhri B, Fiala MA, Shah N, Vij R, Wildes TM . Measuring cardiopulmonary complications of Carfilzomib treatment and associated risk factors using the SEER-Medicare database. Cancer 2020; 126: 808–13. doi: 10.1002/cncr.32601 31721140 PMC6992490

[43] Feng D, Edwards WD, Oh JK, Chandrasekaran K, Grogan M, Martinez MW, et al . Intracardiac thrombosis and embolism in patients with cardiac Amyloidosis. Circulation 2007; 116: 2420–26. doi: 10.1161/CIRCULATIONAHA.107.697763 17984380

[44] Pinder MC, Duan Z, Goodwin JS, Hortobagyi GN, Giordano SH . Congestive heart failure in older women treated with adjuvant anthracycline chemotherapy for breast cancer. J Clin Oncol 2007; 25: 3808–15. doi: 10.1200/JCO.2006.10.4976 17664460

[45] Feher A, Baldassarre LA, Sinusas AJ . Novel cardiac computed tomography methods for the assessment of anthracycline induced cardiotoxicity. Front Cardiovasc Med 2022; 9: 875150. doi: 10.3389/fcvm.2022.875150 35571206 PMC9094702

[46] Kelkar AA, Schultz WM, Khosa F, Schulman-Marcus J, O’Hartaigh BWJ, Gransar H, et al . Long-term prognosis after coronary artery calcium scoring among low-intermediate risk women and men. Circ Cardiovasc Imaging 2016; 9(): e003742. doi: 10.1161/CIRCIMAGING.115.003742 27072301

[47] Hecht HS, Cronin P, Blaha MJ, Budoff MJ, Kazerooni EA, Narula J, et al . SCCT/STR guidelines for coronary artery calcium scoring of Noncontrast noncardiac chest CT scans: A report of the society of cardiovascular computed tomography and society of Thoracic Radiology. J Thorac Imaging 2017; 32: W54–66. doi: 10.1097/RTI.0000000000000287 28832417

[48] Brink JA, Hricak H . Radiology 2040. Radiology 2023; 306: 69–72. doi: 10.1148/radiol.222594 36534608 PMC9792708

[49] Schicchi N, Fogante M, Palumbo P, Agliata G, Esposto Pirani P, Di Cesare E, et al . The sub-Millisievert era in CTCA: the technical basis of the new radiation dose approach. Radiol Med 2020; 125: 1024–39. doi: 10.1007/s11547-020-01280-1 32930945

[50] Nicol M, Baudet M, Cohen-Solal A . Subclinical left ventricular dysfunction during chemotherapy. Card Fail Rev 2019; 5: 31–36. doi: 10.15420/cfr.2018.25.1 30847243 PMC6396067

[51] Meléndez GC, Sukpraphrute B, D’Agostino RB, Jordan JH, Klepin HD, Ellis L, et al . Frequency of left ventricular end-diastolic volume-mediated declines in ejection fraction in patients receiving potentially cardiotoxic cancer treatment. Am J Cardiol 2017; 119: 1637–42. doi: 10.1016/j.amjcard.2017.02.008 28341361 PMC5406277

[52] De Rubeis G, Catapano F, Cundari G, Ascione A, Galea N, Catalano C, et al . Cocaine abuse: an attack to the cardiovascular system-insights from cardiovascular MRI. Radiol Cardiothorac Imaging 2019; 1: e180031. doi: 10.1148/ryct.2019180031 33778503 PMC7970099

[53] Armenian SH, Lacchetti C, Barac A, Carver J, Constine LS, Denduluri N, et al . Preventionand monitoring of cardiac dysfunction in survivors of adult cancers: American society of clinical oncology clinical practice guideline. J Clin Oncol 2017; 35: 893–911. doi: 10.1200/JCO.2016.70.5400 27918725

[54] Messroghli DR, Moon JC, Ferreira VM, Grosse-Wortmann L, He T, Kellman P, et al . Clinical recommendations for cardiovascular magnetic resonance mapping of T1, T2, T2* and extracellular volume: A consensus statement by the society for cardiovascular magnetic resonance (SCMR) endorsed by the European Association for cardiovascular imaging (EACVI). J Cardiovasc Magn Reson 2017; 19(): 75. doi: 10.1186/s12968-017-0389-8 28992817 PMC5633041

[55] Ferreira VM, Schulz-Menger J, Holmvang G, Kramer CM, Carbone I, Sechtem U, et al . Cardiovascular magnetic resonance in nonischemic myocardial inflammation: expert recommendations. J Am Coll Cardiol 2018; 72: 3158–76. doi: 10.1016/j.jacc.2018.09.072 30545455

[56] Thompson RC, Canby RC, Lojeski EW, Ratner AV, Fallon JT, Pohost GM . Adriamycin cardiotoxicity and proton nuclear magnetic resonance relaxation properties. Am Heart J 1987; 113: 1444–49. doi: 10.1016/0002-8703(87)90660-0 3591613

[57] Burrage MK, Ferreira VM . The use of cardiovascular magnetic resonance as an early non-invasive biomarker for cardiotoxicity in Cardio-oncology. Cardiovasc Diagn Ther 2020; 10: 610–24. doi: 10.21037/cdt-20-165 32695641 PMC7369285

[58] Thavendiranathan P, Amir E, Bedard P, Crean A, Paul N, Nguyen ET, et al . Regional myocardial edema detected by T2 mapping is a feature of cardiotoxicity in breast cancer patients receiving sequential therapy with anthracyclines and Trastuzumab. J Cardiovasc Magn Reson 2014; 16(. doi: 10.1186/1532-429X-16-S1-P273

[59] Lustberg MB, Reinbolt R, Addison D, Ruppert AS, Moore S, Carothers S, et al . Early detection of anthracycline-induced cardiotoxicity in breast cancer survivors with T2 cardiac magnetic resonance. Circ Cardiovasc Imaging 2019; 12(): e008777. doi: 10.1161/CIRCIMAGING.118.008777 31060375 PMC6516083

[60] Haslbauer JD, Lindner S, Valbuena-Lopez S, Zainal H, Zhou H, D’Angelo T, et al . CMR imaging Biosignature of cardiac involvement due to cancer-related treatment by T1 and T2 mapping. Int J Cardiol 2019; 275: 179–86. doi: 10.1016/j.ijcard.2018.10.023 30360992

[61] Wassmuth R, Lentzsch S, Erdbruegger U, Schulz-Menger J, Doerken B, Dietz R, et al . Subclinical cardiotoxic effects of anthracyclines as assessed by magnetic resonance imaging-a pilot study. Am Heart J 2001; 141: 1007–13. doi: 10.1067/mhj.2001.115436 11376317

[62] Lightfoot JC, D’Agostino RB Jr, Hamilton CA, Jordan J, Torti FM, Kock ND, et al . Novel approach to early detection of doxorubicin cardiotoxicity by Gadolinium-enhanced cardiovascular magnetic resonance imaging in an experimental model. Circ Cardiovasc Imaging 2010; 3: 550–58. doi: 10.1161/CIRCIMAGING.109.918540 20622140 PMC3068484

[63] Jordan JH, D’Agostino RB Jr, Hamilton CA, Vasu S, Hall ME, Kitzman DW, et al . Longitudinal assessment of concurrent changes in left ventricular ejection fraction and left ventricular myocardial tissue characteristics after administration of cardiotoxic Chemotherapies using T1-weighted and T2-weighted cardiovascular magnetic resonance. Circ Cardiovasc Imaging 2014; 7: 872–79. doi: 10.1161/CIRCIMAGING.114.002217 25273568 PMC4241241

[64] Fallah-Rad N, Lytwyn M, Fang T, Kirkpatrick I, Jassal DS . Delayed contrast Enhancement cardiac magnetic resonance imaging in Trastuzumab induced cardiomyopathy. J Cardiovasc Magn Reson 2008; 10: 5. doi: 10.1186/1532-429X-10-5 18272009 PMC2244612

[65] Lawley C, Wainwright C, Segelov E, Lynch J, Beith J, McCrohon J . Pilot study evaluating the role of cardiac magnetic resonance imaging in monitoring adjuvant Trastuzumab therapy for breast cancer. Asia Pac J Clin Oncol 2012; 8: 95–100. doi: 10.1111/j.1743-7563.2011.01462.x 22369450

[66] Flett AS, Hayward MP, Ashworth MT, Hansen MS, Taylor AM, Elliott PM, et al . Equilibrium contrast cardiovascular magnetic resonance for the measurement of diffuse myocardial fibrosis: preliminary validation in humans. Circulation 2010; 122: 138–44. doi: 10.1161/CIRCULATIONAHA.109.930636 20585010

[67] Meléndez GC, Jordan JH, D’Agostino RB, Vasu S, Hamilton CA, Hundley WG . Progressive 3-month increase in LV myocardial ECV after anthracycline-based chemotherapy. JACC Cardiovasc Imaging 2017; 10: 708–9. doi: 10.1016/j.jcmg.2016.06.006 27544895 PMC7890530

[68] Neilan TG, Coelho-Filho OR, Pena-Herrera D, Shah RV, Jerosch-Herold M, Francis SA, et al . Left ventricular mass in patients with a cardiomyopathy after treatment with anthracyclines. Am J Cardiol 2012; 110: 1679–86. doi: 10.1016/j.amjcard.2012.07.040 22917553 PMC3496816

[69] Dang Y, Hou Y . The Prognostic value of late Gadolinium Enhancement in heart diseases: an umbrella review of meta-analyses of observational studies. Eur Radiol 2021; 31: 4528–37. doi: 10.1007/s00330-020-07437-w 33409800

[70] Nacif MS, Kawel N, Lee JJ, Chen X, Yao J, Zavodni A, et al . Interstitial myocardial fibrosis assessed as extracellular volume fraction with low-radiation-dose cardiac CT. Radiology 2012; 264: 876–83. doi: 10.1148/radiol.12112458 22771879 PMC3426854

[71] Bandula S, White SK, Flett AS, Lawrence D, Pugliese F, Ashworth MT, et al . Measurement of myocardial extracellular volume fraction by using equilibrium contrast-enhanced CT: validation against histologic findings. Radiology 2013; 269: 396–403. doi: 10.1148/radiol.13130130 23878282

[72] Lee H-J, Im DJ, Youn J-C, Chang S, Suh YJ, Hong YJ, et al . Myocardial extracellular volume fraction with dual-energy equilibrium contrast-enhanced cardiac CT in nonischemic cardiomyopathy: A prospective comparison with cardiac MR imaging. Radiology 2016; 280: 49–57. doi: 10.1148/radiol.2016151289 27322972

[73] Monti CB, Zanardo M, Bosetti T, Alì M, De Benedictis E, Luporini A, et al . Assessment of myocardial extracellular volume on body computed tomography in breast cancer patients treated with anthracyclines. Quant Imaging Med Surg 2020; 10: 934–44. doi: 10.21037/qims.2020.04.05 32489918 PMC7242290

[74] Hong YJ, Kim TK, Hong D, Park CH, Yoo SJ, Wickum ME, et al . Myocardial characterization using dual-energy CT in doxorubicin-induced DCM: comparison with CMR T1-mapping and histology in a rabbit model. JACC Cardiovasc Imaging 2016; 9: 836–45. doi: 10.1016/j.jcmg.2015.12.018 27236517

[75] Soschynski M, Hagen F, Baumann S, Hagar MT, Weiss J, Krauss T, et al . High temporal resolution dual-source photon-counting CT for coronary artery disease: initial multicenter clinical experience. JCM 2022; 11: 6003. doi: 10.3390/jcm11206003 36294324 PMC9604695

[76] Rahman ZU, Sethi P, Murtaza G, Virk HUH, Rai A, Mahmod M, et al . Feature tracking cardiac magnetic resonance imaging: A review of a novel non-invasive cardiac imaging technique. World J Cardiol 2017; 9: 312–19. doi: 10.4330/wjc.v9.i4.312 28515849 PMC5411965

[77] Thavendiranathan P, Zhang L, Zafar A, Drobni ZD, Mahmood SS, Cabral M, et al . Myocardial T1 and T2 mapping by magnetic resonance in patients with immune Checkpoint inhibitor-associated myocarditis. J Am Coll Cardiol 2021; 77: 1503–16. doi: 10.1016/j.jacc.2021.01.050 33766256 PMC8442989

[78] Drafts BC, Twomley KM, D’Agostino R, Lawrence J, Avis N, Ellis LR, et al . Low to moderate dose anthracycline-based chemotherapy is associated with early noninvasive imaging evidence of Subclinical cardiovascular disease. JACC Cardiovasc Imaging 2013; 6: 877–85. doi: 10.1016/j.jcmg.2012.11.017 23643285 PMC3745801

[79] Ong G, Brezden-Masley C, Dhir V, Deva DP, Chan KKW, Chow C-M, et al . Myocardial strain imaging by cardiac magnetic resonance for detection of Subclinical myocardial dysfunction in breast cancer patients receiving Trastuzumab and chemotherapy. Int J Cardiol 2018; 261: 228–33. doi: 10.1016/j.ijcard.2018.03.041 29555336

[80] McDonagh TA, Metra M, Adamo M, Gardner RS, Baumbach A, Böhm M, et al . ESC guidelines for the diagnosis and treatment of acute and chronic heart failure. developed by the task force for the diagnosis and treatment of acute and chronic heart failure of the European society of cardiology (ESC). with the special contribution of the heart failure Association (HFA) of the ESC. G Ital Cardiol (Rome) 2021. 10.1016/j.rec.2022.05.005 35636830

[81] Houbois CP, Nolan M, Somerset E, Shalmon T, Esmaeilzadeh M, Lamacie MM, et al . Serial cardiovascular magnetic resonance strain measurements to identify cardiotoxicity in breast cancer: comparison with echocardiography. JACC Cardiovasc Imaging 2021; 14: 962–74. doi: 10.1016/j.jcmg.2020.09.039 33248962

[82] Feola M, Garrone O, Occelli M, Francini A, Biggi A, Visconti G, et al . Cardiotoxicity after anthracycline chemotherapy in breast carcinoma: effects on left ventricular ejection fraction, troponin I and brain natriuretic peptide. Int J Cardiol 2011; 148: 194–98. doi: 10.1016/j.ijcard.2009.09.564 19945181

[83] Jordan JH, Sukpraphrute B, Meléndez GC, Jolly M-P, D’Agostino RB Jr, Hundley WG . Early myocardial strain changes during potentially cardiotoxic chemotherapy may occur as a result of reductions in left ventricular end-diastolic volume: the need to interpret left ventricular strain with volumes. Circulation 2017; 135: 2575–77. doi: 10.1161/CIRCULATIONAHA.117.027930 28630272 PMC5508602

[84] Childs AC, Phaneuf SL, Dirks AJ, Phillips T, Leeuwenburgh C . Doxorubicin treatment in vivo causes cytochrome C release and cardiomyocyte apoptosis, as well as increased mitochondrial efficiency, superoxide dismutase activity, and Bcl-2:BAX ratio. Cancer Res 2002; 62: 4592–98. 12183413

[85] Sørensen BS, Sinding J, Andersen AH, Alsner J, Jensen PB, Westergaard O . Mode of action of Topoisomerase II-targeting agents at a specific DNA sequence. uncoupling the DNA binding, cleavage and Religation events. J Mol Biol 1992; 228: 778–86. doi: 10.1016/0022-2836(92)90863-f 1335085

[86] Kim Y, Ma A-G, Kitta K, Fitch SN, Ikeda T, Ihara Y, et al . Anthracycline-induced suppression of GATA-4 transcription factor: implication in the regulation of cardiac Myocyte apoptosis. Mol Pharmacol 2003; 63: 368–77. doi: 10.1124/mol.63.2.368 12527808

[87] Ganame J, Claus P, Eyskens B, Uyttebroeck A, Renard M, D’hooge J, et al . Acute cardiac functional and morphological changes after anthracycline infusions in children. Am J Cardiol 2007; 99: 974–77. doi: 10.1016/j.amjcard.2006.10.063 17398195

[88] Ferreira de Souza T, Quinaglia A C Silva T, Osorio Costa F, Shah R, Neilan TG, Velloso L, et al . Anthracycline therapy is associated with cardiomyocyte atrophy and Preclinical manifestations of heart disease. JACC Cardiovasc Imaging 2018; 11: 1045–55. doi: 10.1016/j.jcmg.2018.05.012 30092965 PMC6196358

[89] Jordan JH, Castellino SM, Meléndez GC, Klepin HD, Ellis LR, Lamar Z, et al . Left ventricular mass change after anthracycline chemotherapy. Circ Heart Fail 2018; 11(): e004560. doi: 10.1161/CIRCHEARTFAILURE.117.004560 29991488 PMC6729136

[90] Cadour F, Cautela J, Rapacchi S, Varoquaux A, Habert P, Arnaud F, et al . Cardiac MRI features and Prognostic value in immune Checkpoint inhibitor-induced myocarditis. Radiology 2022; 303: 512–21. doi: 10.1148/radiol.211765 35230185

[91] Neilan TG, Rothenberg ML, Amiri-Kordestani L, Sullivan RJ, Steingart RM, Gregory W, et al . Myocarditis associated with immune Checkpoint inhibitors: an expert consensus on data gaps and a call to action. Oncologist 2018; 23: 874–78. doi: 10.1634/theoncologist.2018-0157 29802220 PMC6156187

[92] Thavendiranathan P, Negishi T, Somerset E, Negishi K, Penicka M, Lemieux J, et al . Strain-guided management of potentially cardiotoxic cancer therapy. J Am Coll Cardiol 2021; 77: 392–401. doi: 10.1016/j.jacc.2020.11.020 33220426

[93] Lyon AR, Dent S, Stanway S, Earl H, Brezden-Masley C, Cohen-Solal A, et al . Baseline cardiovascular risk assessment in cancer patients scheduled to receive cardiotoxic cancer therapies: a position statement and new risk assessment tools from the Cardio-oncology study group of the heart failure Association of the European society of cardiology in collaboration with the International Cardio-oncology society. Eur J Heart Fail 2020; 22: 1945–60. doi: 10.1002/ejhf.1920 32463967 PMC8019326

[94] Koutroumpakis E, Xu T, Lopez-Mattei J, Pan T, Lu Y, Irizarry-Caro JA, et al . Coronary artery calcium score on standard of care oncologic CT scans for the prediction of adverse cardiovascular events in patients with non-small cell lung cancer treated with concurrent Chemoradiotherapy. Front Cardiovasc Med 2022; 9: 1071701. doi: 10.3389/fcvm.2022.1071701 36531700 PMC9755726

[95] Feuchtner GM, Plank F, Beyer C, Barbieri F, Widmann G, Spitaler P, et al . Cardiac computed tomography: state of the art and future horizons. J Clin Med 2022; 11(): 4429. doi: 10.3390/jcm11154429 35956045 PMC9369220

[96] Francone M, Figliozzi S, Monti L, Loewe C, Catapano F . Multiparametric cardiac magnetic resonance unveiling the mechanisms and early manifestations of anticancer drug cardiotoxicity. European Radiology 2023. doi: 10.1007/s00330-023-09948-8 37464110

